# MiR-142-3p is a Critical Modulator of TNF-mediated Neuronal Toxicity in Multiple Sclerosis

**DOI:** 10.2174/1570159X21666230404103914

**Published:** 2023-09-25

**Authors:** Francesca De Vito, Sara Balletta, Silvia Caioli, Alessandra Musella, Livia Guadalupi, Valentina Vanni, Diego Fresegna, Mario Stampanoni Bassi, Luana Gilio, Krizia Sanna, Antonietta Gentile, Antonio Bruno, Ettore Dolcetti, Fabio Buttari, Luigi Pavone, Roberto Furlan, Annamaria Finardi, Emerald Perlas, Eran Hornstein, Diego Centonze, Georgia Mandolesi

**Affiliations:** 1 Unit of Neurology, IRCCS Neuromed, Pozzilli, Isernia, Italy;; 2 Department of Systems Medicine, Tor Vergata University, Rome, Italy;; 3 Synaptic Immunopathology Lab, IRCCS San Raffaele Roma, Rome, Italy;; 4 Department of Human Sciences and Quality of Life Promotion University of Rome San Raffaele, Rome, Italy;; 5 Neuroimmunology Unit, Institute of Experimental Neurology (INSpe), Division of Neuroscience, San Raffaele Scientific Institute, Milan, Italy;; 6 Mouse Biology Unit, European Molecular Biology Laboratory, Monterotondo Scalo, Rome, Italy;; 7 Department of Molecular Genetics, Weizmann Institute of Science, Rehovot, Israel

**Keywords:** Multiple sclerosis, experimental autoimmune encephalomyelitis, microRNA, neuroinflammation, synaptopathy, biological marker

## Abstract

**Background:**

TNF-dependent synaptotoxicity contributes to the neuronal damage occurring in patients with Multiple Sclerosis (pwMS) and its mouse model Experimental Autoimmune Encephalomyelitis (EAE). Here, we investigated miR-142-3p, a synaptotoxic microRNA induced by inflammation in EAE and MS, as a potential downstream effector of TNF signalling.

**Methods:**

Electrophysiological recordings, supported by molecular, biochemical and histochemical analyses, were performed to explore TNF-synaptotoxicity in the striatum of EAE and healthy mice. MiR-142 heterozygous (miR-142 HE) mice and/or LNA-anti miR-142-3p strategy were used to verify the TNF-miR-142-3p axis hypothesis. The cerebrospinal fluid (CSF) of 151 pwMS was analysed to evaluate possible correlation between TNF and miR-142-3p levels and their impact on clinical parameters (*e.g*. progression index (PI), age-related clinical severity (gARMSS)) and MRI measurements at diagnosis (T0).

**Results:**

High levels of TNF and miR-142-3p were detected in both EAE striatum and MS-CSF. The TNF-dependent glutamatergic alterations were prevented in the inflamed striatum of EAE miR-142 HE mice. Accordingly, TNF was ineffective in healthy striatal slices incubated with LNA-anti miR-142-3p. However, both preclinical and clinical data did not validate the TNF-miR-142-3p axis hypothesis, suggesting a permissive neuronal role of miR-142-3p on TNF-signalling. Clinical data showed a negative impact of each molecule on disease course and/or brain lesions and unveiled that their high levels exert a detrimental synergistic effect on disease activity, PI and white matter lesion volume.

**Conclusion:**

We propose miR-142-3p as a critical modulator of TNF-mediated neuronal toxicity and suggest a detrimental synergistic action of these molecules on MS pathology.

## 1 INTRODUCTION

Early degenerative changes of the grey matter (GM) contribute to disease progression and motor and cognitive deficits in MS, a chronic inflammatory and demyelinating disease of the central nervous system (CNS) [1-3]. Several preclinical and clinical studies have identified diffuse synaptic dysfunction and loss as early hallmarks of MS pathophysiology, occurring even independently of axonal demyelination and transection [4-6]. Coherently, studies both in MS and in the EAE mouse model, revealed that pro-inflammatory cytokines, such as Interleukin-1β (IL-1β) and Tumor Necrosis Factor (TNF), released from infiltrating T cells and from activated microglia and astroglia, participate in the early imbalance between glutamate excitatory and GABA inhibitory transmission, leading to hyperexcitability and excitotoxic neuronal damage in several brain areas [7, 8]. Pharmacological and non-pharmacological interventions aiming at repairing inflammatory synaptic dysfunction are particularly appealing since synaptopathy is an early and reversible process. However, reliable biomarkers are still missing and the mechanisms at the basis of inflammation-driven synaptopathy are largely elusive.

TNF is emerging as a pro-inflammatory and principal mediator in MS pathogenesis. Notably, high intrathecal TNF levels have been associated with disease progression and correlated with early GM damage [3, 9-11]. Such an effect is likely due to the TNF-direct synaptotoxic action that leads to excitotoxic damage in both MS and EAE models. We previously demonstrated a TNF-dependent enhancement of glutamatergic transmission in the EAE striatum that leads to synaptic loss [12]. Accordingly, we observed that administration of TNF in healthy conditions, *in vitro* or *in vivo*, was able to promote synaptotoxic effects in the striatum, mimicking the glutamatergic alterations observed in EAE [12, 13]. Furthermore, we showed that high levels of TNF circulating in the cerebrospinal fluid (CSF) of progressive pwMS cause excitotoxicity and neuronal swelling in striatal slices derived from healthy mice (chimeric *ex-vivo* MS model) [14]. Since the molecular mechanisms underlying TNF-synaptopathy are still elusive, in the present work, we sought to investigate a possible link with microRNA-142-3p (miR-142-3p), a microRNA that we found highly expressed in the EAE brain and in the CSF of active pwMS [15, 16]. We suggested that this miRNA is directly involved in MS and EAE pathogenesis as a crucial regulator of both CNS and immune system. Notably, miR-142 knockout (KO) mice are fully resistant to EAE induction [15], while EAE miR-142 heterozygous mice (miR-142 HE), which are responsive to EAE induction, are more sensitive to a currently used MS disease modifying therapy, with respect to their EAE WT littermates. Furthermore, we observed that in the EAE cerebellum, miR-142-3p was highly expressed in inflammatory lesions (where lymphocytes, microglia and glial cells predominate) and caused glutamate synaptic dysfunction, in response to IL-1β, by targeting the glial glutamate-aspartate transporter (GLAST). Accordingly, the IL-1β-dependent enhancement of cerebellar glutamatergic transmission was recovered in EAE miR-142 HE mice or in EAE WT mice treated with locked nucleic acid (LNA)-inhibitor of miR-142-3p [15, 16]. Notably, in the CSF of pwMS miR-142-3p levels correlate with IL-1β levels, with a worse Progression Index (PI) at diagnosis and increased excitability [16]. In this study, we explored a potential link between miR-142-3p and TNF-synaptotoxicity to better clarify their involvement in both EAE and MS pathology. We analysed three experimental conditions: the mouse striatum under EAE condition, control striatal slices under TNF stimulation, and the CSF of pwMS for correlation analysis. As a primary readout of TNF-synaptotoxicity, we looked at the enhancement of the glutamatergic kinetic properties recorded from medium spiny neurons (MSNs), and used miR-142 heterozygous mice and LNA-anti miR-142-3p strategy to investigate the link between TNF-dependent synaptopathy and miR-142-3p.

## 2 MATERIALS AND METHODS

### 2.1 Mice and EAE Induction

Female C57BL/6 mice (carrying a heterozygous insertion of a LacZ gene trap in the miR-142 locus (HE^+/-^; [17] and relative wild-type (WT^+/+^) littermates were used for all the experiments (Plaisant, Rome, Italy). Animal experiments described in this study were conducted according to the guidelines set by the Internal Institutional Review Committee, the European Directive 2010/63/EU and the European Recommendations 526/2007 and the Italian D. Lgs 26/2014. Mice were housed under constant conditions in an animal facility with a regular 12 h light/dark cycle. Food and water were supplied ad libitum. Chronic-progressive EAE was induced in eight- to ten-week-old mice by active immunization with an emulsion of myelin oligodendrocyte glycoprotein peptide 35-55 (MOG_35-55_) in Complete Freund’s Adjuvant (CFA), followed by intravenous administration of pertussis toxin (500 ng) twice (at days 0 and 2) as previously described [15, 16, 18]. EAE clinical score was recorded daily according to a 0-5 scale. For each animal, the onset day was recorded as the day post-immunization (dpi) when it showed the first clinical manifestations (score > 0). EAE mice were compared with untreated mice (Ctrl mice). Details of EAE symptoms evaluation are provided in the Supplementary materials.

### 2.2 Electrophysiology

Mice (11-13 wks) were killed by cervical dislocation and the brains were removed. Then, corticostriatal coronal slices (200 µm) were cut by means of a vibratome (Leica VT1200 - Leica biosystems, Wetzlar, Germany) and transferred to a recording chamber with continuously flowing artificial CSF (ACSF) (34°C, 2-3 mL/min) gassed with 95% O_2_-5% CO_2_. Whole-cell patch-clamp recordings of glutamate-mediated spontaneous excitatory postsynaptic currents (sEPSCs) were made with borosilicate glass pipettes (1.8 mm outer diameter; 2-4 MΩ), in voltage-clamp mode, at the holding potential of -80 mV. Only data from putative MSNs [19,20], which account for over 95% of the entire population of striatal neurons, were included in this study. In some experiments, corticostriatal slices derived from HE mice were incubated for 2 hours with TNF (0.6 µM in PBS-BSA 0.1%; Peprotech) plus AM281 (2 µM; Tocris) or vehicle (DMSO 0.02%). The same experiments without incubation with TNF were performed in EAE HE mice. To lower the miR-142-3p levels, some corticostriatal slices derived from control mice were pre-incubated with LNA anti-miR-142-3p for 30 minutes and then with TNF plus LNA anti-miR-142-3p for 2 hours, in a chamber containing ACSF, before electrophysiological recordings. LNA scramble was used instead of LNA anti-miR-142-3p as control condition. After incubation, brain slices were used for electrophysiological experiments or were decorticated and stored at -80°C until use for Real Time PCR and Western blot analysis. In the experiments performed to evaluate miR-142-3p neuronal role in the TNF-mediated effect, LNA anti-miR-142-3p (0.5 pmol/µl), or LNA scramble (0.5 pmol/µl), were added in the pipette-intracellular solution used to record the sEPSCs in MSNs of control corticostriatal slices following the incubation with TNF (0.6 µM; 2 hours). For each parameter analysed, one to six cells per animal were recorded. Two to five animals per group were used. All details are provided in Supplementary Materials.

### 2.3 Real Time PCR (qPCR)

Whole striata were dissected from control and EAE mice in the acute phase of the disease (20-25 dpi) in RNAse-free conditions and stored at -80°C until used. Total RNA was extracted as previously described [15] and the expression of miR-142-3p and Tnf, Aif1, Cd3e and Gfap mRNAs was evaluated using TaqMan technology. For miR-142-3p detection, miR-142-3p TaqMan miRNA assay and TaqMan miRNA Reverse Transcription Kit (Applied Biosystems) or miRCURY LNA miRNA SYBR^®^ Green PCRs (QIAGEN) were used according to the manufacturer’s instructions. For mRNA detection, about 500 ng of total RNA was reverse-transcribed using High-Capacity cDNA Reverse Transcription Kit (Applied Biosystem) according to the manufacturer's instructions. Then, 6-20 ng of cDNA were amplified and each reaction of amplification was performed in triplicates with SensiMix II Probe Kit (Bioline, Meridian Life Science) by using the Applied Biosystem 7900HT Fast Real Time PCR system and data were represented as 2^-ΔΔCt^. U6B snRNA and β-actin were used as endogenous control for miR-142-3p and mRNA normalisation, respectively. For *ex-vivo* experiments, the evaluation of miR-142-3p levels was performed on corticostriatal slices (200 μm) derived from naïve WT mice, incubated for 2 hours with TNF (0.6 µM in PBS-BSA 0.1%; Peprotech) and then, decorticated. MiR-142-3p detection and qPCR data analysis were performed as for whole striata. qPCR primers used are listed in Supplementary Table **S1**.

### 2.4 RNA *in situ* Hybridization (ISH)

Striata were collected, fresh frozen in OCT, and sectioned at 20 µm onto Superfrost Plus slides. ISH was performed using LNA probes complementary to miR-142-3p and labelled with digoxigenin at both 3 and 5 ends as (QIAGEN) [15].

### 2.5 Western Blot (WB) Analysis

Whole striata dissected from control and EAE mice in the acute phase of the disease (20-25 dpi) were homogenised in RIPA buffer plus protease inhibitor mixture (Sigma) and sonicated. After sonication, the homogenates were centrifuged at 13000×g for 20 min and the supernatant was collected. Protein content was quantified according to the BCA Assay method (Thermofisher). Five μg of striatal extract were loaded onto a sodium dodecyl-sulfate polyacrylamide gel. Gel was blotted onto PVDF membrane (Millipore). WB experiments were performed as previously described [15, 18] and the following primary antibodies were used: mouse anti- β-actin (1:20000, Sigma) for 1 h room temperature (RT); mouse anti-GluR1 (1:1000, Millipore). Membranes were incubated with a secondary HRP-conjugated IgG anti-mouse (1:10000, 1 h RT, Abcam). Results were presented as data normalised to β-actin and Ctrl-WT values N = 4-5 per group.

### 2.6 Immunofluorescence and Confocal Microscopy

Mice were deeply anesthetized and intracardially perfused with ice-cold 4% paraformaldehyde (PFA) at 21-25 dpi (N = 2-3 per group). Collected brains were post-fixed in 4% PFA for 2 h and equilibrated with 30% sucrose for at least one night. Thirty-micrometer-thick coronal sections were serially cut on a frozen microtome including the whole striatum to perform immunofluorescence experiments as previously described [18]. The following primary antibodies were used overnight at 4°C in Triton X-100 0.25%: rabbit anti-Iba1 (1:750, Wako), mouse anti-TNF (1:1500, Abcam), rabbit anti-GFAP (1:500, Dako), rat anti-CD3 (1:300, Biorad). After being washed, sections were incubated with the secondary antibody Alexa-488 conjugated donkey anti-Rabbit or anti-Rat (1:200, Invitrogen); Cy3-conjugated donkey anti-mouse (1:200, Jackson) for 2 h at RT. Nuclei were stained with DAPI (1 μl/ml; Sigma Aldrich). Images were acquired using a Nikon Eclipse TI2 confocal laser-scanner microscope with 20x, 40x and 60x objectives and were processed using ImageJ software. Details of images acquisition and analysis are reported in the Supplementary Materials.

### 2.7 Patients’ Recruitment and Clinical Parameters

All subjects who were enrolled in this retrospective study provided their written informed consent according to the Declaration of Helsinki principles and the Institutional Review Board. For details on demographic and clinical characteristics (Table **1**). Further details are provided in Supplementary Materials.

### 2.8 RNA Extraction from CSF and miR-142-3p Detection

CSF withdrawal was performed within 24 hours from patients’ admittance. Sample preparation and qPCR experiments were performed as described in [16, 21]. MiR-204-5p was used as the endogenous reference for the ΔCt calculation (Ct miR-142-3p - Ct miR-204-5p). Low ΔCt-values indicate high miR-142-3p levels and data were presented as 2^(- ΔCt). The median value of CSF miR-142-3p levels (0.010 relative quantification to miR-204-5p) in the cohort of all pwMS was used as a cut-off to divide patients into Low miR-142-3p patients’ and High-miR-142-3p patients’ groups.

### 2.9 Detection of TNF in the CSF

Bio-Plex multiplex assay (Bio-Rad Laboratories, Hercules, CA, USA) was used for the quantification of TNF [21]. Proteins below the detection sensitivity of the standard curve were considered as 0 pg/ml. The median value of CSF TNF levels (2.250 pg/ml) in the cohort of all pwMS was used as a cut-off to divide patients into Low-TNF patients’ and High-TNF patients’ groups.

## 3 EXPERIMENTAL

Statistical analysis was performed with Prism GraphPad 6.0 and IBM SPSS Statistics 17.0. Data distribution was tested for normality by using Kolmogorov-Smirnov test and Shapiro-Wilk test. Correlation between TNF, miR-142-3p CSF levels and demographic and clinical parameters were made by nonparametric Spearman correlation analysis. Differences between the two groups were analysed using two-tailed Paired or Unpaired Student’s t-test or Mann-Whitney test, as appropriate. Multiple comparisons were performed by ANOVA followed by Tukey’s HSD. Data were presented as the mean ± S.E.M, unless otherwise specified. The significance level was established at *p* <0.05. The exact statistical test used for each experiment and its details can be found in the figure legends and Supplementary Table **S2**.

## 4 RESULTS

### 4.1 Inflammatory Enhancement of Striatal Glutamatergic Transmission is Impaired in EAE miR-142-HE Mice

**Fig. (1):**
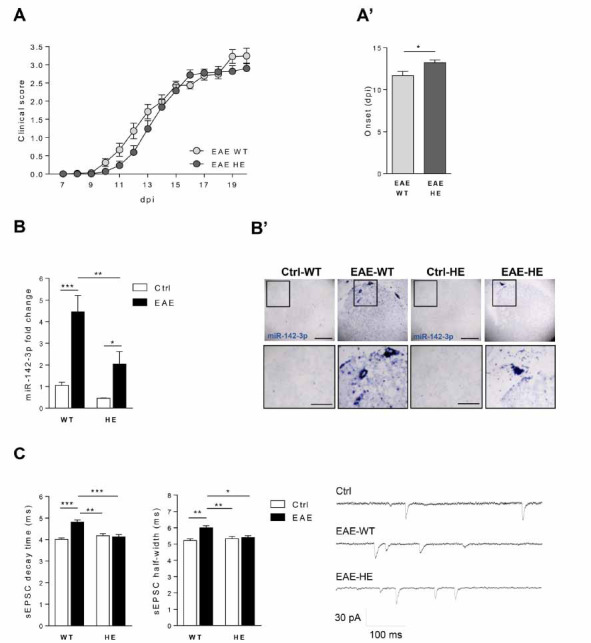
EAE striatal glutamatergic enhancement is impaired in miR-142-HE mice. (**A**) Time course of clinical score in EAE-WT and EAE-HE mice. No significant differences were observed between the two experimental groups (EAE-WT: N = 32; EAE-HE: N = 29); statistical analysis by Two-way ANOVA followed by Bonferroni’s post-hoc test. (**A’**) The clinical manifestation of motor symptoms was delayed in EAE-HE mice compared to EAE-WT (EAE-WT: N = 32; EAE-HE: N = 29; Unpaired T-test). (**B**) Reduced levels of miR-142-3p were detected by qRT-PCR in EAE-HE striata (Ctrl-WT: N = 8; EAE-WT: N = 5; Ctrl-HE: N = 9; EAE-HE: N = 7). All data were normalized to U6B by ΔΔCt calculation (mean SEM *vs.* controls); statistical analysis by One-way ANOVA followed by Tukey’s post hoc test; (**B’**) Representative image of ISH with miR-142-3p probe in coronal striatal sections of EAE-WT and EAE-HE mice and relative controls. MiR-142-3p was more pronounced in EAE-WT compared to EAE-HE striatum. The signal was almost undetectable in control animals (Ctrl-WT, EAE-WT, Ctrl-HE, EAE-HE: N = 4). Low magnification, scale bar 100 µm. High magnification, scale bar 50 µm. (**C**) Whole-cell patch-clamp recordings from MSNs of EAE-WT and EAE-HE corticostriatal slices and relative controls. Histograms reporting the sEPSC kinetics in the four experimental groups. In a control condition, WT and HE striatal MSNs showed similar sEPSC values. Although EAE induction significantly increased sEPSC kinetics in WT, it did not affect decay time and half width in HE mice (21-28 dpi; Ctrl-WT, n =17; EAE-WT, n = 10; Ctrl-HE, n = 24; EAE-HE, n = 18); statistical analysis by One-way ANOVA followed by Tukey’s post hoc test. Right, representative sEPSC traces were obtained from Ctrl, EAE-WT and EAE-HE mice. (**A-C**) Data are expressed as mean ± SEM; *p <0.05; **p <0.01; ***p <0.001; N represents the numbers of mice and n represents the numbers of cells.

We previously observed that EAE miR-142 HE mice, during the acute phase of the disease were protected from enhancement of the IL-β-dependent glutamatergic transmission, at least in the inflamed cerebellum [16]. Here, we characterized the EAE striatum of miR-142 HE mice and their wild type littermates to investigate the involvement of miR-142-3p in the TNF-dependent synaptic dysfunctions typical of this brain region. To this aim, we induced MOG 35-55 EAE in miR-142 HE and their WT littermates . As previously demonstrated [16], the disease severity was similar between the two experimental groups (EAE-WT and EAE-HE: *p >*0.05, Fig. **1A**). Here, we observed that EAE HE mice showed one day delay in the appearance of disease symptoms compared with WT mice (EAE-WT *vs* EAE-HE: *p <*0.05, Fig. **1A’**), suggesting that miR-142 3p levels might influence the disease onset. Quantification of striatal miR-142-3p (20 dpi; motor disability score ≥ 1) by qPCR analysis revealed that miR-142-3p levels were increased in both genotypes following EAE induction (Ctrl-WT *vs.* EAE-WT, *p <*0.001; Ctrl-HE *vs.* EAE-HE, *p <*0.05; Fig. **1B**) and, in line with the HE miR-142 genotype, miRNA levels were significantly lower in EAE miR-142-HE relative to EAE WT mice (EAE-WT *vs.* EAE-HE: *p <*0.01). Consistently, in the ISH experiments the staining of miR-142-3p was more prominent in the striatum of EAE WT compared to EAE miR-142-HE (Fig. **1B**’). Next, we evaluated by means of patch-clamp recordings whether the reduced miR-142-3p levels in miR-142-HE striatum could influence glutamatergic neurotransmission. EAE striatal synaptopathy is characterized by an enhanced duration of the glutamatergic transmission at the level of MSNs, presenting a slower decay phase and higher half width of the spontaneous excitatory synaptic currents (sEPSC), leading to excitotoxic glutamate-mediated damage [12, 14]. Notably, in the EAE miR-142-HE striatum, we observed full recovery of sEPSC kinetic alterations, during the acute phase of the disease (18-28 dpi), in comparison to EAE WT group (Fig. **1C**). In particular, the values of decay time and half width of EAE miR-142 HE mice were unchanged with respect to controls (Ctrl-HE *vs.* EAE-HE: *p >*0.05) and were significantly lower than those recorded from EAE WT MSNs (decay time: EAE-WT *vs.* EAE-HE: *p <*0.001; half width: EAE-WT *vs.* EAE-HE: *p <*0.05). No differences were observed in terms of sEPSC amplitude between EAE miR-142-HE and EAE WT mice (*p >*0.05). Control WT and HE mice did not show any difference in terms of striatal glutamatergic kinetic properties (decay time and half width: Ctrl-WT *vs.* Ctrl-HE: *p >*0.05), indicating that a reduced expression of miR-142-3p did not impact normal spontaneous glutamatergic transmission.

These results suggest that reduced miR-142-3p levels in EAE miR-142-HE striatum are protective against the EAE-mediated synaptopathy.

### 4.2 GluR1 Expression is Modulated in the Inflamed Striatum of EAE miR-142-HE Mice

**Fig. (2):**
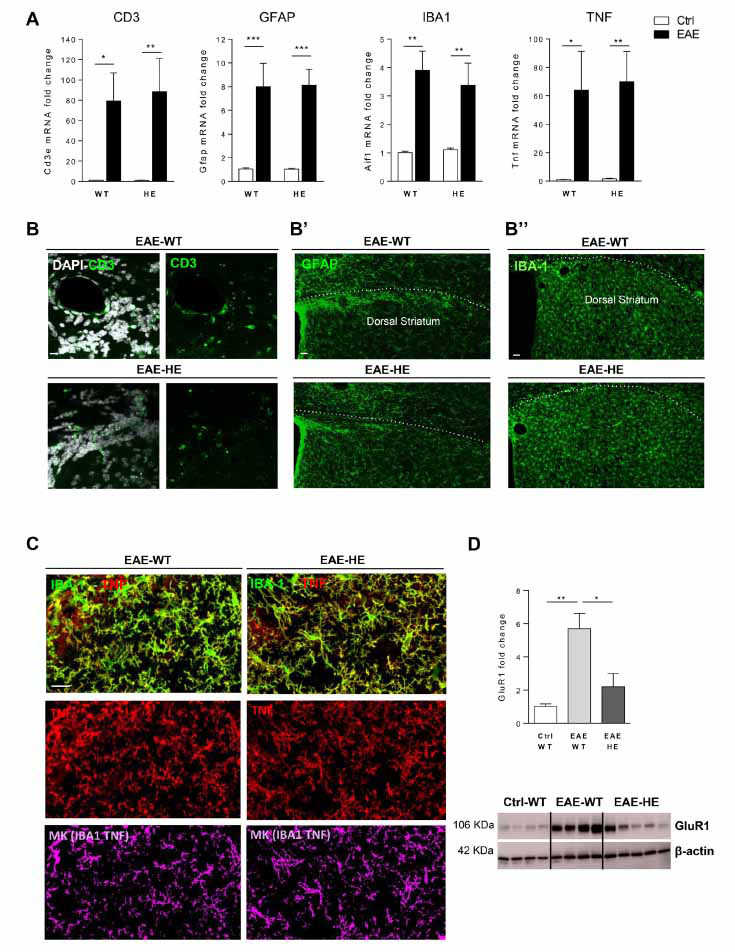
GluR-1 levels are reduced in the EAE-HE striatum in the presence of neuroinflammation. (**A**) qRT-PCR analysis showed no changes in the levels of Tnf, Aif1, Cd3e and Gfap mRNAs between EAE-HE and EAE-WT striata (Ctrl-WT: N = 8; EAE-WT: N = 5; Ctrl-HE: N = 9; EAE-HE: N = 7). All data were normalised by ΔΔCt calculation to β-actin and Ctrl-WT; statistical analysis by One-way ANOVA followed by Tukey’s post hoc test. B-B”, Representative confocal microscopy images of corticostriatal sections from EAE-WT and EAE-HE mice (21-25 dpi) detecting CD3^+^ infiltrating T cells (green fluorescence) (**B**) GFAP^+^ astroglial cells (green fluorescence) (**B’**), IBA1^+^ microglia/macrophages cells (green fluorescence) (**B”**) as inflammatory markers upregulated in EAE. The local striatal neuroinflammation was similar between EAE-WT and EAE-HE mice. Scale bars were IBA1, GFAP: 40µm; CD3 20µm; (n = 3-4 per mouse; N = 2-3 per group). (**C**) represented confocal microscopy images of corticostriatal sections from EAE-WT and EAE-HE mice (21-25 dpi) showing TNF (red fluorescence) double staining with IBA1 (green fluorescence). The colocalization mask for TNF and IBA1 signal suggests that the TNF production from microglia was comparable between EAE-WT and EAE-HE mice. (Scale bars: 20 µm; n = 3-4 per mouse; N = 2-3 per group) (**D**) Representative western blot images and quantification of GluR1 protein levels in striatal slices from EAE-WT and EAE-HE mice relative to Ctrl-WT. The expression of GluR1 subunit was significantly reduced in EAE-HE mice compared to EAE-WT (Ctrl-WT: N = 4, EAE-WT: N = 4, EAE-HE: N = 5 mice per group; Unpaired T-test). All data were normalised to β-actin and Ctrl-WT. (**A-D**) Data are expressed as mean ± SEM; *p <0.05; **p <0.01; ***p <0.001; N represents the numbers of mice and n represents the numbers of slices.

Considering that the changes of sEPSC kinetics properties in EAE striatum have been ascribed to neuroinflammation and, more specifically, to TNF released by resident and non-resident immune cells [12, 13], we analysed the expression of the main neuroinflammatory markers by qPCR. As shown in Fig. (**2A**), the mRNA levels of *Tnf*, *Aif1* (to assess microglia activation), *Cd3e* (to assess T lymphocyte infiltration) and *Gfap* (to assess astroglia activation) were significantly increased following EAE induction in both mouse genotypes (*Cd3e*: Ctrl-WT *vs.* EAE-WT, *p <*0.05; Ctrl-HE *vs.* EAE-HE, *p <*0.01; *Gfap*: Ctrl-WT *vs.* EAE-WT, *p <*0.001; Ctrl-HE *vs.* EAE-HE, *p <*0.001; *Aif1*: Ctrl-WT *vs.* EAE-WT, *p <*0.01; Ctrl-HE *vs.* EAE-HE, *p <*0.01; *Tnf*: Ctrl-WT *vs.* EAE-WT, *p <*0.05; Ctrl-HE *vs.* EAE-HE, *p <*0.01). Notably, the inflammatory status was indistinguishable between EAE WT and EAE miR-142-HE conditions (*Cd3e*, *Gfap*, *Aif1*, *Tnf*: EAE-WT *vs.* EAE-HE, *p >*0.05; Fig. **2A**), although miR-142-HE mice were protected from the EAE-induced synaptic damage. Coherently with qPCR results, qualitative immunostaining experiments suggest a similar expression of the neuroinflammatory markers in EAE WT and EAE miR-142-HE striata (Fig. **2B-B”**). In particular, the TNF production from microglial cells was similar between the two experimental groups, as highlighted by the colocalization mask in Fig. (**2C**).

Since aberrant AMPA receptor expression is involved in EAE glutamatergic transmission abnormalities, we measured GluR1 subunit level in total striata from EAE WT and EAE miR-142-HE mice, to assess a possible impact of a reduced miR-142-3p expression on TNF signal cascade [12]. As expected, western blot analysis showed that the protein level of GluR1 AMPA subunit was significantly increased following EAE induction (Ctrl-WT *vs.* EAE-WT: *p <*0.01), but it was significantly lower in the EAE miR-142-HE striata compared to the EAE WT samples (EAE-WT *vs.* EAE-HE: *p <*0.05; Fig. **2D**). No difference was observed in control conditions (data not shown).

Altogether, these data indicate that reduced miR-142-3p levels in the EAE striatum are protective in terms of AMPA-dependent glutamate excitotoxicity, even in the presence of EAE neuroinflammation.

### 4.3 MiR-142-HE Striatal Slices are not Responsive to TNF Stimulation *in vitro*

**Fig. (3):**
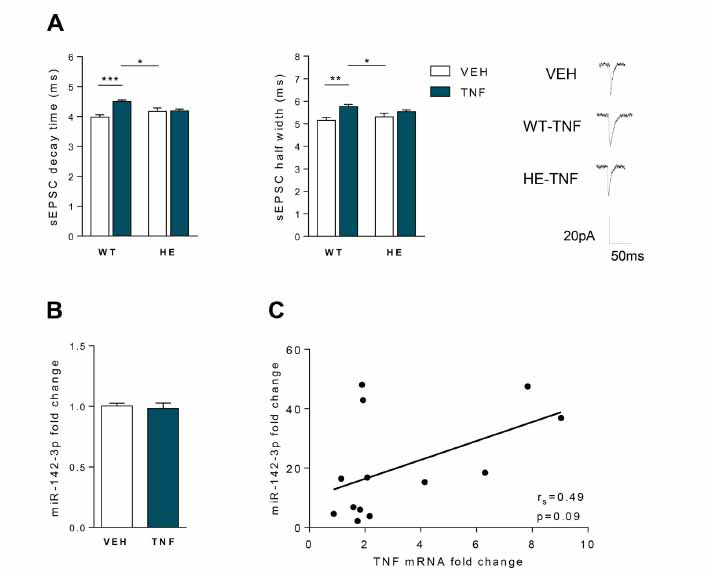
The healthy striatum of miR-142-HE mice is protected from TNF-dependent enhancement of glutamatergic transmission. (**A**) Whole-cell patch-clamp recordings from MSNs of WT and HE corticostriatal slices incubated with TNF (0.6 µM) or vehicle (VEH, PBS-BSA 0.1%) for 2 hours. Histograms reporting the sEPSC kinetics in the four experimental groups. TNF incubation significantly increased decay time and half width in WT, but failed to affect these kinetic parameters in HE conditions (WT-VEH, n = 14; WT-TNF, n = 21; HE-VEH, n = 20; HE-TNF, n = 12); statistical analysis by One-way ANOVA followed by Tukey’s post hoc test. Right, representative sEPSC mean peaks of the different experimental conditions. (**B**) qRT-PCR analysis showed no changes in the levels of miR-142-3p in WT striatal slices following TNF incubation (0.6 µM, 2 hours) (WT-VEH: n = 14; WT-TNF: n = 14; Unpaired T-test). All data were normalised to U6B by ΔΔCt calculation (mean SEM *vs.* VEH). (**C**) Correlation plot between miR-142-3p (2^-^ΔΔCt^ relative to U6B) and TNF (2^-^ΔΔCt^ relative to actine) levels in the striatum of EAE mice. No correlation was observed (N = 13, Spearman’s r = 0.49, *p >*0.05). (**A-C**) Data are expressed as mean ± SEM; *p <0.05; **p <0.01; ***p <0.001; N represents numbers of mice and n represents the numbers of cells or number of slices.

On the basis of the above results and previous findings in EAE cerebellum [15], we hypothesised that EAE striatal synaptopathy could be mediated by an up-regulation of miR-142-3p induced by TNF. To address this hypothesis, we took advantage of a consolidated *in vitro* approach based on acute incubation of healthy striatal slices with TNF, resulting in an enhancement of glutamatergic transmission similar to EAE condition [12]. Corticostriatal slices derived from naïve WT and miR-142-HE mice were incubated with TNF (0.6 µM) or vehicle (VEH) for 2 hours to perform both electrophysiological and qPCR experiments. As shown in Fig. (**3A**), TNF failed to enhance the sEPSC kinetic properties in miR-142-HE striatal slices (decay time and half width: HE-VEH *vs.* HE-TNF: *p >*0.05), whereas it induced the expected increase of the duration of the glutamatergic transmission in WT slices relatively to the basal conditions (WT-TNF *vs.* WT-VEH: decay time *p <*0.001 and half width *p <*0.01; WT-TNF *vs.* HE-VEH: decay time and half width *p <*0.05), in accordance with the *in vivo* experiments [13]. Notably, the quantification of miR-142-3p levels by qPCR in WT striatal slices revealed that incubation of TNF was not able to upregulate the expression of this miRNA (*p >*0.05; Fig. **3B**). Consistently, we did not observe any correlation between miR-142-3p and TNF levels in the striatum of EAE mice (N = 13, r_s_ = 0,489, *p* > 0.05, Fig. **3C**).

These results indicate that miR-142-HE striatal slices are not functionally responsive to TNF stimulation and suggest that TNF effects in WT and likely in EAE striatal slices are not associated with miR-142-3p upregulation.

### 4.4 TNF-dependent Striatal Synaptopathy is Influenced by Neuronal miR-142-3p Levels

**Fig. (4):**
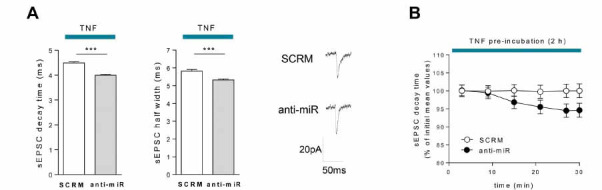
LNA anti-miR-142-3p treatment impairs striatal TNF-mediated synaptopathy. (**A**) Whole-cell patch-clamp recordings from MSNs of WT corticostriatal slices pre-incubated (30 minutes) with LNA anti-miR-142-3p (anti-miR) or scramble (SCRM) and subsequently incubated with TNF (0.6 µM) for 2 hours. Histograms reporting that the TNF effect on kinetic parameters (decay time and half width) was significantly lowered by anti-miR-142-3p compared to scramble (SCRM-TNF, n = 28; anti-miR-TNF, n = 20; Unpaired T-test); Right, Representative sEPSC mean peaks of the two experimental conditions. (**B**) Whole-cell patch-clamp recordings from MSNs of WT corticostriatal slices incubated with TNF (0.6 µM) for 2 hours. Anti-miR or SCRM was added into the pipette solution. Anti-miR neuronal application induced a reduction of the decay time over the time compared to its initial value (n = 9; 0-5 *vs.* 25-30, *p <*0.05; Paired T-test). (**A** and **B**) Data are expressed as mean ± SEM; ***p < 0.001; N represents the number of mice and n represents the number of cells.

To exclude a potential effect derived from the transgenic nature of miR142-HE mice, we performed electrophysiological recordings in WT corticostriatal slices preincubated with an LNA-based miR-142-3p inhibitor (anti-miR) or LNA scramble (SCRM) for 30 minutes before adding TNF in bath for 2 hours. Consistently with what was observed in HE mice, TNF was unable to induce significant increases of glutamatergic activity in anti-miR condition, whereas it promoted the expected excitotoxic synaptopathy in the scramble condition (decay time and half width: SCRM-TNF *vs.* anti-miR-TNF: *p <*0.0001; Fig. **4A**). These results clearly proved the existence of a causal link between the failure of the synaptotoxic effect of TNF and low expression of miR-142-3p, suggesting a permissive role for this miRNA in the TNF-signalling cascade.

To investigate a possible role for miR-142-3p at neuronal level, we studied the effect of a local reduction of miR-142-3p in MSNs on TNF-driven glutamatergic synaptic dysfunctions, by adding anti-miR-142-3p LNA in the pipette solution. Interestingly, the intracellular inhibition of miR-142-3p, obtained after 30 minutes of anti-miR spreading through the neuronal cytoplasm, was enough to significantly reduce the alteration of sEPSC decay time triggered by TNF (0-5 min *vs.* 25-30 min: *p <*0.05; Paired t test; Fig. **4B**). No changes were observed for half width (0-5 min *vs.* 25-30 min: *p <*0.05; Paired t test; not shown). This specific neuronal action on TNF-induced synaptopathy was not observed in MSNs receiving the LNA scramble, highlighting, for the first time, a possible synaptic function of miR-142-3p in neurons.

These results indicate that TNF needs adequate levels of miR-142-3p to induce striatal synaptic dysfunctions, suggesting a neuronal permissive role of miR-142-3p.

### 4.5 TNF-mediated Synaptopathy is Impaired in miR-142-HE Mice Independently of CB1 Receptor Signalling

**Fig. (5):**
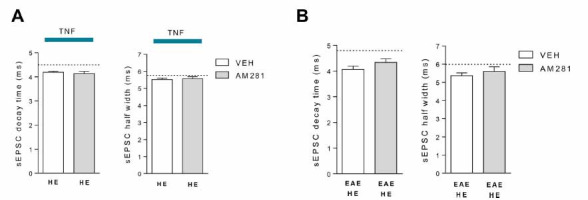
The rescue of the TNF-dependent glutamatergic enhancement is independent of CB1R activation in miR-142-HE striatum. (**A**) Whole-cell patch-clamp recordings from MSNs of miR-142-HE corticostriatal slices incubated with TNF plus vehicle or with TNF plus AM281 for 2 hours, respectively. Histograms showing that the kinetic parameters (decay time and half width) were not altered even following the blockade of CB1R (HE-TNF-VEH, n =10; HE-TNF-AM281, n = 20); statistical analysis by unpaired T-test. Dotted lines indicate the mean values of sEPSC decay time and half width obtained from WT corticostriatal slices incubated with TNF. (**B**) Whole-cell patch-clamp recordings from MSNs of EAE miR-142-HE corticostriatal slices incubated with vehicle or with AM281 for 2 hours, respectively. Histograms showing that the kinetic parameters (decay time and half width) were not altered even following the blockade of CB1R (EAE-HE-VEH, n = 14; EAE-HE-AM281, n = 9); statistical analysis by unpaired T-test. Dotted lines indicate the mean values of sEPSC decay time and half width obtained from EAE-WT slices. (**A** and **B**) Data are expressed as mean ± SEM; N represents the number of mice and n represents the number of cells.

To further investigate the possible mechanisms underlying TNF and miR-142-3p interplay in glutamatergic synaptic pathology, we addressed our attention on the endocannabinoid system and its link with TNF-dependent synaptopathy. We previously demonstrated that type-1 cannabinoid receptor (CB1R) stimulation was able to prevent TNF-mediated alteration of striatal sEPSCs [22]. In order to evaluate whether the failure of kinetic alteration observed in miR-142-HE TNF-striatum was due to an overactivation of the endocannabinoid system, we performed electrophysiological experiments with AM281 to block the CB1R. In particular, we incubated corticostriatal slices derived from naïve miR-142-HE mice with TNF plus AM281 for 2 hours. As shown in Fig. (**5A**), the blockade of the CB1R in HE condition was not able to restore the TNF-dependent glutamatergic kinetic alterations (decay time and half width: *p >* 0.05) typically observed in naïve WT slices [22]. Similar results were observed when AM281 was incubated on EAE miR-142-HE striatal slices (Fig. **5B**).

Overall these data suggest that striatal glutamatergic transmission in miR-142-HE mice is preserved from TNF-induced synaptopathy through a mechanism independent of CB1R activation.

### 4.6 High Levels of Both TNF and miR-142-3p in the CSF of pwMS are Associated with an Ongoing Disease Activity Likely Detrimental for MS Disease Course

To translate our preclinical data into human pathology, we investigated the association between TNF and miR-142-3p levels in the CSF of pwMS, and potential correlations with other variables relevant to disease course in pwMS.

**Fig. (6):**
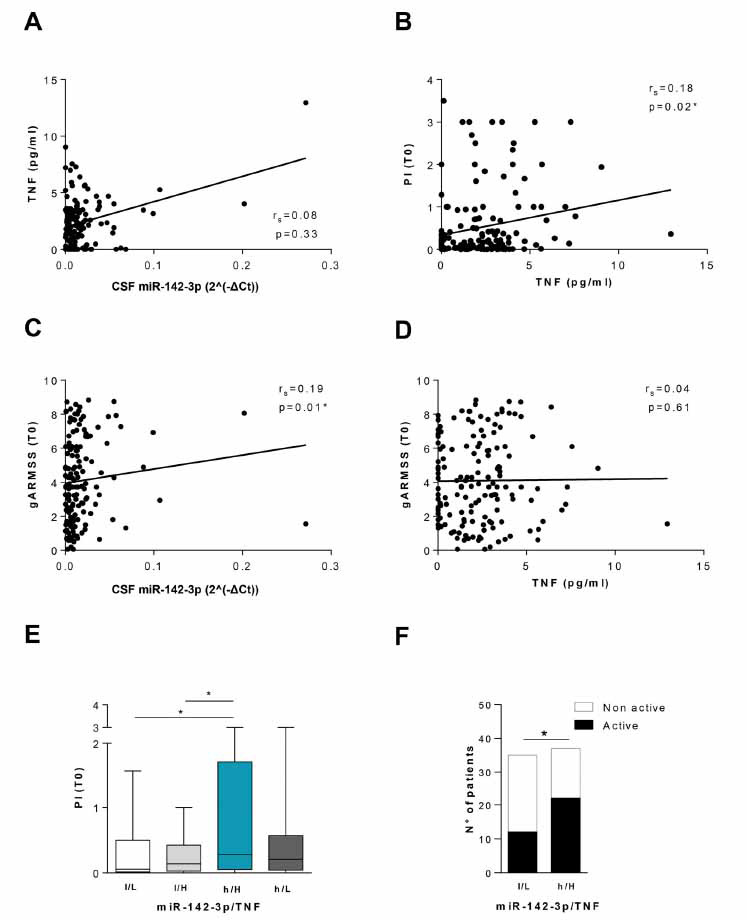
High CSF levels of TNF and miR-142-3p are associated with an unfavourable MS disease progression. (**A**) Correlation plot between CSF TNF (pg/mL) and CSF miR-142-3p levels (2^(- ΔCt) relative to miR-204-5p) at T0. No correlation was observed (n = 151, Spearman’s r = 0.079, *p >*0.05). (**B**) A positive correlation was observed between CSF TNF levels (pg/mL) and the Progression Index at the withdrawal (PI(T0)) (n = 151, Spearman’s r = 0.184, *p <*0.05). (**C**) CSF miR-142-3p levels directly correlate with the global ARMSS (gARMSS) score at T0 (n = 151, Spearman’s r = 0.19, *p <*0.05). (**D**) Correlation plot between CSF TNF levels (pg/mL) and the gARMSS score at T0. No correlation was observed (n = 151, Spearman’s r = 0.04, *p >*0.05). (**E**) Box-and-whisker plots of the Progression Index at the withdrawal (PI(T0)) in patients with low miR-142-3p and low TNF levels (l/L, n = 40), low miR-142-3p and high TNF levels (l/H, n = 35), high miR-142-3p and high TNF levels (h/H, n = 39), high miR-142-3p and low TNF levels (h/L, n = 37). The h/H patients’ group presented at T0 a worse PI relative to the l/L and l/H groups (Mann Whitney test, *p <*0.05). Median values of miR-142-3p and TNF were used as respective thresholds. (**F**) Histograms showing the number of active and non-active pwMS of the low miR-142-3p and low TNF levels group (l/L, n = 40) and high miR-142-3p and high TNF levels group (h/H, n = 39). The number of active pwMS was significantly higher in the h/H group compared to the l/L group (Contingency analysis, *p <*0.05).

We performed Bio-Plex multiplex assay in a cohort of 151 pwMS (clinically isolated syndrome, CIS, n = 18; RRMS, n = 108; PMS, n = 25) to quantify CSF TNF levels. MiR-142-3p levels were previously quantified by qPCR in the same cohort of patients [16]. In accordance with preclinical results (Fig. **3B-C**), we did not observe any correlation between TNF and miR-142-3p levels (n = 151, r_s_ = 0.079, *p >* 0.05; Fig. **6A**).

Next, we evaluated possible correlations between each molecule and demographic parameters (sex, age) or clinical features (Table **1**) recorded at the time of CSF withdrawal (T0).

No correlations with demographic parameters, EDSS and disease duration were observed (data not shown). Interestingly, we revealed a positive correlation between CSF TNF levels and progression index estimated at the time of diagnosis (PI (T0) (n = 151, r_s_ = 0.184, *p <*0.05; Fig. **6B**). Notably, in the same cohort of patients we previously observed that high CSF miR-142-3p levels were associated with a worse disease progression [16]. We also evaluated the global Age Related Multiple Sclerosis Severity (gARMSS) score, obtained by ranking EDSS values based on the patient’s age at the time of diagnosis [23]. A positive correlation was found between miR-142-3p levels and gARMSS (r_s_= 0.190, *p < 0.05, Fig. **6C**), while TNF levels did not correlate with this clinical parameter (r_s_ = 0.040, *p* > 0.05, Fig. **6D**). We then explored a possible synergistic effect of these molecules on the disease course by evaluating the PI (T0) and gARMSS (T0) distributions according to the combined CSF levels of TNF and miR-142-3p. To this aim, we divided our cohort of pwMS in four subgroups by using the median value of both CSF miR-142-3p and TNF levels as cut-offs (miR-142-3p: 0.010 relative to miR-204-5p; TNF: 2.250 pg/ml). PI (T0) values were significantly higher in the high-miR-142-3p/ High-TNF (h/H) group compared to the other groups (low-miR-142-3p/Low-TNF (l/L) and low miR-142-3p/High-TNF (l/H); *p <*0.05; Fig. **6E**), except for the high miR-142-3p/ Low-TNF group (h/H *vs.* h/L *p >*0.05). We did not find any difference of PI (T0) between the low-miR-142-3p/ High-TNF (l/H) and the low-miR-142-3p/Low-TNF (l/L) groups (*p >*0.05). PwMS with high CSF levels of miR-142-3p but reduced amount of TNF (h/L), showed an intermediate PI (T0) between h/H and l/H subjects (*p >*0.05), suggesting a driving effect of inflammatory miR-142-3p levels in determining the MS course. Regarding the gARMSS (T0) parameter, the score of the high-miR-142-3p/High-TNF(h/H) group was higher in comparison to the low miR-142-3p/High-TNF group (l/H) (*p <*0.05), while no differences were observed in comparison with the other groups (*p* > 0.05 data no shown). Finally, the negative impact of miR-142-3p and TNF on the clinical course was confirmed by the evidence that the ratio between the number of active and non-active pwMS was significantly higher in the high-miR-142-3p/High-TNF group (h/H, n=39) compared to the low-miR-142-3p/Low-TNF group (l/L, n = 40) (Contingency analysis, *p* < 0.05, Fig. **6F**).

These clinical results strengthen our previous observation of a negative impact of miR-142-3p on disease course and strongly suggest that patients carrying high levels of both TNF and miR-142-3p present a worse disease activity and course.

### 4.7 A Prominent Brain Damage at the Time of Diagnosis is Present in pwMS with high CSF Levels of TNF and miR-142-3p

To evaluate the role of CSF levels of miR-142-3p and TNF on brain damage, we investigated MRI structural measurements in a subgroup of 39 pwMS (Table **2**).

**Fig. (7):**
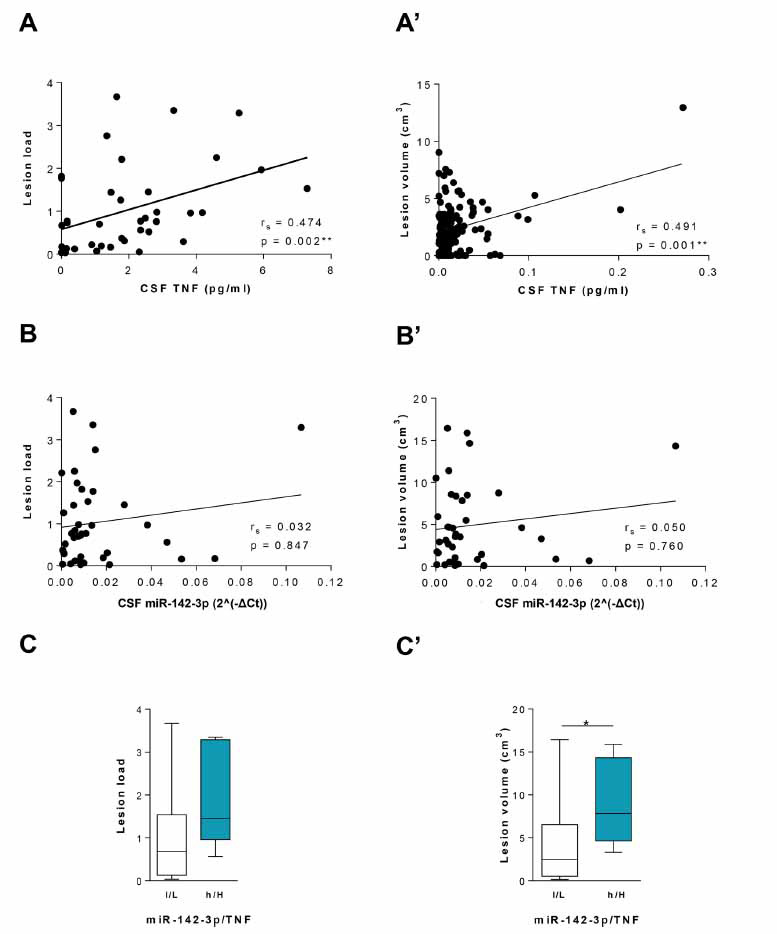
MRI structural measurements in MS patients. (**A** and **A’**) Positive correlations were revealed between CSF TNF levels (pg/mL) with both lesion load (n = 39, Spearman’s r = 0.474, *p <*0.01) and lesion volume (cm^3^) (n = 39, Spearman’s r = 0.491, *p <*0.01). (**B** and **B’**) No correlations were found between CSF miR-142-3p levels with both lesion load (n = 39, Spearman’s r = 0.032, *p >*0.05) and lesion volume (cm^3^) (n = 39, Spearman’s r = 0.050, *p >*0.05). (**C** and **C’**) Box-and-whisker plots of the cortical lesion load (**C**) and lesion volume (cm^3^) (**C’**) in patients with low-miR-142-3p and low-TNF levels (l/L, n = 14) and high-miR-142-3p and high-TNF levels (h/H, n = 7). The h/H patients’ group showed a significantly increased lesion volume (cm^3^) relative to the l/L group (Mann Whitney test, *p <*0.05) (**C’**).

We observed a positive correlation of CSF TNF levels with both white matter lesion load (r_s_ = 0.474, *p <*0.01, Fig. **7A**) and lesion volume (r_s_ = 0.491, *p <*0.01, Fig. **7A**’). Conversely, no correlations were found between CSF miR-142-3p levels and the white matter lesion load or lesion volume (r_s_ = 0.032, *p >* 0.05, Fig. **7B**; r_s_ = 0.05, *p >*0.05, Fig. **7B**’). Notably, in the subgroup of patients with high levels of miR-142-3p and TNF (h/H, n = 7), the lesion volume parameter was significantly higher compared to the low-miR-142-3p/Low-TNF group (l/L, n = 14, *p <*0.05, Fig. **7C**), although no difference was observed in terms of lesion load (*p >*0.05; Fig. **7C’**).

Altogether these data further highlight a link between TNF, miR-142-3p and brain lesions in MS, and strongly suggest that the brain damage induced by neuroinflammatory milieu in pwMS might depend on a concomitant action of miR-142-3p and TNF.

## 5 DISCUSSION

In the present work, we explored by means of preclinical and clinical observations the interaction between TNF and miR-142-3p, two synaptotoxic molecules highly involved in MS- and EAE- GM pathology. We revealed that low levels of miR-142-3p can prevent the enhancement of the excitatory synaptic transmission induced by TNF in the striatum, a brain region particularly affected in both EAE and MS. It emerged that the interaction between TNF and miR-142-3p does not engage a regulatory axis in which miR-142-3p is induced by TNF to alter synaptic transmission, but rather a miR-142-3p permissive action on TNF-signalling. Furthermore, since elevated levels of both miR-142-3p and TNF at diagnosis seem to be a signature of disease activity as well as of a worse disease course and white matter damage, we suggest the potentiality of future personalised therapies possibly against both TNF and miR-142-3p to dampen inflammatory neurotoxic effects in MS. TNF is a proinflammatory cytokine that exerts both homeostatic and pathophysiological roles in the CNS. Several data clearly show that TNF modulates glutamatergic neurotransmission in the striatum and other brain areas [24-29]. During neuroinflammation, intermingled loops involving infiltrating lymphocytes, microglia, astroglia and neuronal cells have been proposed to explain the relationship between TNF and excitotoxicity [30]. TNF mainly released by activated microglia can potentiate glutamate-mediated cytotoxicity by two complementary mechanisms: indirectly, by inhibiting glutamate transport on astrocytes, and directly, by rapidly triggering the surface expression of calcium permeable AMPARs (composed of GluR1 subunits) and NMDA receptors, while decreasing inhibitory GABAA receptors on neurons [30]. In the EAE model, striatal glutamatergic synaptic changes have been associated with an abnormal microglial release of this cytokine, with an increased expression and phosphorylation of AMPARs and with a loss of dendritic spines [12]. Interestingly, intracerebroventricular (icv) injection of TNF was able to enhance striatal glutamatergic transmission, mimicking the synaptic alterations observed during EAE [13]. Such modifications in striatal excitatory neurotransmission were paralleled by behavioural abnormalities that could be reversed by the icv administration of etanercept, an anti-TNF drug [13]. It has been proposed that synaptic hyperexcitability in EAE follows TNF-induced up-scaling, a negative feedback response induced by synaptic hypo-stimulation in response to brain damage [31]. Notably, in a CSF chimeric *ex-vivo* MS model, we observed that CSF taken from active pwMS (but not from no-active or control subjects) was able to exacerbate the glutamatergic transmission recorded in mouse corticostriatal slices, in a TNF-dependent manner. Pre-treatment with etanercept blocked indeed the ability of MS-derived CSF to induce synaptic alterations [14]. Notably, similar results were also obtained in the T-cell chimeric *ex-vivo* MS model [32]. Altogether these data clearly indicate that during MS, TNF released by immune cells might be sufficient to induce a diffuse glutamatergic synaptopathy [31] and the long-lasting effects of such phenomenon can be detrimental for brain function and integrity.

In this work, we hypothesised miR-142-3p as a putative mediator of the TNF-dependent enhancement of the glutamatergic transmission in the striatum, considering our previous observations that linked IL-1β to miR-142-3p and synaptic dysfunction in both EAE and MS [15]. Similarly to the EAE cerebellum, we detected high striatal levels of miR-142-3p in both WT and miR-142-HE mice during the acute phase of disease, with an intense localization likely in the inflammatory lesions. Notably, in the presence of an inflamed striatum, we observed that the enhancement of the sEPSC kinetic properties as well as of the AMPAR GluR1 levels, were prevented in EAE miR-142-HE mice, showing values similar to healthy conditions. Accordingly, the incubation of TNF on striatal slices derived from healthy miR-142-HE was ineffective in inducing glutamatergic synaptic alterations. Interestingly, when we performed qPCR analysis we did not detect a TNF-dependent upregulation of miR-142-3p, differently from what we observed in the cerebellum under neuroinflammatory conditions [15, 16]. To exclude possible effects related to the transgenic nature of miR-142 HE mice, we incubated LNA-anti miR-142-3p on striatal slices and could still observe an amelioration of TNF-induced kinetic enhancement. This experiment clearly demonstrates a possible interference of miR-142-3p on TNF-synaptopathy, in particular when physiological levels of miR-142-3p are downregulated. In support of a possible local role of miR-142-3p in the MSNs we conducted electrophysiological experiments by infusing LNA-anti-miR142-3p through the patch pipette and we observed a partial synaptic recovery of the glutamatergic alterations induced by TNF in comparison to LNA-scramble. This experiment was quite challenging since the patch-recording experimental condition did not allow either a pre-infusion of LNA-anti-miR through the patch pipette before TNF incubation, nor a prolonged recording to detect an efficient effect of the LNA-anti-miR intracellular treatment. Nevertheless, these results were supported by the other experiments with bath application of the LNA-anti-miR providing a full protective effect from TNF treatment.

The molecular mechanism underlying such a ‘permissive role’ of endogenous miR-142-3p to TNF signalling needs further investigation. The regulation of mRNAs directly or indirectly targeted by miR-142-3p may potentially act at different levels. We assume that physiological levels of miRNA-142-3p may guarantee a proper TNF-signalling at MSNs by acting through different potential mechanisms: i) by maintaining the delicate balance between the soluble and transmembrane forms of TNF, TNFRI and TNFRII, in favor of TNF/TNFRI signalling [33]; ii) by regulating the endocytosis of AMPARs [34]; iii) by interfering with neuroprotective signalling that is able to counteract the TNF-dependent glutamate enhancement. In this regard, we investigated the neuroprotective action of CB1R whose activation blocks the TNF-dependent increase in surface AMPARs and protects neurons from excitotoxicity both *in vitro* [35] and *in vivo* [22]. We previously demonstrated that the glutamatergic alterations in the EAE striatum were remarkably more evident in CB1R-KO EAE mice and that stimulation of CB1Rs (by HU210) fully prevented TNF-synaptotoxicity in healthy striatal slices [22]. Coherently, application of CB1R antagonist (AM281) prevented the HU210 effect on TNF-mediated sEPSC duration. Here, we supposed an enhancement of CB1 signalling in miR-142-HE mice. However, incubation of AM281 on miR-142-HE corticostriatal striatal slices failed to restore TNF-glutamatergic enhancement. Further experiments are warranted to clarify the molecular mechanisms linking miR-142-3p and TNF, including the hypothesis that TNF could modify the miRNA cargo of astrocyte shed extracellular vesicles to potentially regulate synaptic signalling in neurons [36].

On the basis of these preclinical results that suggest a potential permissive role of miR-142-3p on TNF signalling we moved to the MS context and analysed possible interactions between these two molecules. According to preclinical results, we did not detect any correlation between the CSF levels of miR-142-p and TNF in a large cohort of pwMS. We then evaluated the impact of each molecule or their synergistic effects on clinical and MRI measurements.

Regarding TNF, the correlation analysis with clinical and MRI measurements strongly support previous observations on the potential use of cytokines as biomarkers for MS [37, 38]. Here, we found a positive but weak correlation between TNF levels and PI (T0) and no correlation with the gARMSS score (T0). Notably, a strong correlation between WM lesion load and lesion volume emerged in the subgroup of patients at the time of diagnosis. Previous observations from other investigators showed elevated levels of CSF TNF in pwMS with a high cortical lesion load at diagnosis, as well as, increased global cortical thickness and levels of NF-L in the CSF [39]. Of note, in a large cohort of MS patients, the same investigators showed that CSF levels of TNF and sTNF-R1 at the time of diagnosis correlated with new white and grey matter lesions, increased EDSS score, and evidence of disease activity after 2 years [3].

Regarding miR-142-3p, our previous observations showed that miR-142-3p levels in the CSF of pwMS positively correlate with PI (T0) and neuronal excitability [16]. Here, we provide evidence of a significant correlation between CSF miR-142-3p levels and gARMSS, indicating that pwMS with high amounts of miR-142-3p at diagnosis present a worse disability ranked by age. Although these observations were not implemented by clinical measurements at follow-up, PI (T0) and gARMSS evaluated in a large cohort of naive-to-DMT patients provide a valid evaluation of MS severity at diagnosis. In a subgroup of patients, the WM lesion load and lesion volume did not correlate with miR-142-3p levels, suggesting that the CSF levels of miR-142-3p are not strictly indicative of WM damage. However, we cannot exclude that miR-142-3p is highly expressed in the white matter lesions of pwMS, as reported in literature [40].

Interestingly, by stratifying pwMS in accordance with TNF and miR-142-3p levels in the CSF, we proposed the existence of a synergy between the two molecules that can influence disease course and pathology. An association indeed emerged between high levels of miR-142-3p and TNF (h/H) and the active phase of the disease as well as unfavourable PI (T0), suggesting a synergistic action that may lay down in multiple synaptotoxic effects at the time of diagnosis. Notably, in the subgroup of patients with MRI parameters, the WM lesion volume significantly increased in the presence of high levels of both molecules. Altogether these data suggest that the disease course of pwMS carrying high levels of both molecules at diagnosis might be severe from the onset and likely worse at the follow-up.

## CONCLUSION

The present study provides a better knowledge of TNF-signalling and miR-142-3p role in the CNS and reveals a synergistic contribution of these molecules to EAE- and MS-brain pathology. Regardless of the underlying mechanisms, we propose miR-142-3p as a critical modulator of TNF-mediated neuronal excitotoxicity in neuroinflammatory conditions. Overall, our previous and current findings indicate that the interplay between miR-142-3p and inflammatory cytokines is complex depending on their local and temporal expression but their early detection in the CSF of pwMS is not only important for MS prognosis but also to establish personalised therapies that potentially target miR-142-3p.

## Figures and Tables

**Table 1:** Demographic and clinical characteristics of all pwMS at CSF withdrawal (T0).

**Variable**	**All Patients**	**Patients’ Groups**			
		**l/L**	**l/H**	**h/H**	**h/L**
N	151	40	35	39	37
Gender (F/M)	97/54	26/14	23/12	25/14	23/14
Age (yr)	39.08 ± 12.27	40.55 ± 11.38	40.67 ± 13.64	35.79 ± 11.10	39.44 ± 12.82
MS Subtypes: non-PMS	126	7/25	2/27	6/27	4/28
MS Subtypes: PMS	25	7/1	4/2	4/2	4/1
Disease activity (y/n/NA)	75/61/15	12/23/5	18/12/5	22/15/2	23/11/3
CSF oligoclonal banding (y/n/NA)	119/23/9	34/5/1	27/5/3	27/8/4	31/5/1
Disease duration(mos)	12.430(2.400-39.270)	20.250(3.789-79.700)	12.430(2.930-35.500)	5.430(1.070-37.130)	13.230(2.780-38.220)
EDSS(score 0-10)	2.000(1.000-3.000)	2.000(1.000-3.375)	1.500(1.000-3.000)	2.000(1.000-3.000)	2.000(1.000-3.000)
PI(T0)(EDSS score/mos)	0.162(0.028-0.682)	0.058(0.013-0.498)	0.136(0.027-0.423)	0.279(0.053-1.714)	0.206(0.043-0.570)
gARMSS (T0)	3.730(1.900-6.090)	3.730(1.638-6.610)	3.270(1.500-4.800)	4.290(2.710-6.840)	4.100(2.020-5.900)
TNF (pg/ml)	2.250(0.780-3.350)	0.900(0.030-1.598)	3.100(2.820-3.620)	3.510(3.100-4.340)	0.660(0.000-1.790)
CSF miR-142-3p(2^^(- ΔCt)^ rel. to miR-204-5p)	0.010(0.004-0.020)	0.004(0.001-0.006)	0.004(0.001-0.006)	0.020(0.013-0.038)	0.018(0.012-0.027)

**Note:** Age is mean ± standard deviation. Other data are median and 25^th^-75^th^ percentiles. All patients have been considered and divided in Low miR-142-3p/Low TNF group (l/L), Low miR-142-3p/High-TNF group (l/H), High-miR-142-3p/High-TNF group (h/H) and High miR-142-3p/Low TNF group (h/L). **Abbreviations**: Non-PMS = Patients with Clinically Isolated Syndrome (CIS), Relapsing-Remitting MS (RRMS); PMS = Patients with Primary Progressive MS (PPMS), Secondary Progressive MS (SPMS); Disease activity: clinical and/or neuroradiological signs of inflammatory brain lesions; CSF = Cerebrospinal Fluid; EDSS = Expanded Disability Status Scale; PI(T0) = Progression Index at T0; gARMSS(T0) = global Age Related Multiple Sclerosis Severity; TNF = Tumor Necrosis Factor. F = female; M = male; yr = year; y/n/NA = yes/no/Not Available; mos = months.

**Table 2:** Demographic and clinical characteristics of a subgroup of pwMS at CSF withdrawal (T0).

**Variable**	**MS Patients**
N(CIS/RRMS/PMS)	39(5/27/7)
Gender (F/M)	24/15
Age (yr)	38.86±12.48
Disease activity (y/n)	17/22
CSF oligoclonal banding (y/n/NA)	31/7/1
Disease duration(mos)	8.0(1.230-37.100)
EDSS (score 0-10)	2.000(1.000-3.000)
CSF miR-142-3p(2^^(- ΔCt)^ rel. to miR-204-5p)	0.008(0.005-0.010)
PI(T0)(EDSS score/mos)	0.161(0.032-0.500)
gARMSS (T0)	3.730(2.080-6.090)
CSF TNF(pg/ml)	1.760(0.170-2.82)

**Note:** Age is mean ± standard deviation. Other data are median and 25^th^-75^th^ percentiles. **Abbreviations:** Clinically isolated syndrome (CIS), Relapsing-remitting MS (RRMS), progressive MS (PMS). CSF = Cerebrospinal Fluid; EDSS = Expanded Disability Status Scale; PI(T0) = Progression Index at T0; gARMSS(T0) = global Age Related Multiple Sclerosis Severity; TNF = Tumor Necrosis Factor. F = female; M = male; mos = months; y/n/NA = yes/no/Not Available; yr = year; IL-1β/IL-1ra ratio = ratio between Interleukin-1β and Interleukin-1β receptor antagonist.

## Data Availability

The datasets presented in this study can be found in online repositories: https://repository.neuromed.it/index.php/f/25164.

## LIST OF ABBREVIATIONS

CNS: Central Nervous System
CSF: Cerebrospinal Fluid
GM: Grey Matter
LNA: Locked Nucleic Acid
MSNs: Medium Spiny Neurons
sEPSC: Spontaneous Excitatory Synaptic Currents
TNF: Tumor Necrosis Factor
