# Supplementary material for: MiR-142-3p is a Critical Modulator of TNF-mediated Neuronal Toxicity in Multiple Sclerosis
Source: Curr Neuropharmacol. 2023 Sep 25;21(12):2567–82. doi: 10.2174/1570159X21666230404103914 (PMC10616916; doi:10.2174/1570159X21666230404103914)
Supplement: Supplementary file 1 [file CN-21-2567_SD1.pdf]

Supplementary Material

MiR-142-3p is a Critical Modulator of TNF-mediated Neuronal Toxicity in Multiple Sclerosis

Francesca De Vito[1,#], Sara Balletta[1,2,#], Silvia Caioli[1], Alessandra Musella[3,4], Livia Guadalupi[2,3], Valentina Vanni[3], Diego Fresegna[3], Mario Stampanoni Bassi[1], Luana Gilio[1], Krizia Sanna[2], Antonietta Gentile[3], Antonio Bruno[1,2], Ettore Dolcetti[1,2], Fabio Buttari[1], Luigi Pavone[1], Roberto Furlan[5], Annamaria Finardi[5], Emerald Perlas[6], Eran Hornstein[7], Diego Centonze[1,2,*] and Georgia Mandolesi[3,4]

[1]Unit of Neurology, IRCCS Neuromed, Pozzilli, Isernia, Italy; [2]Department of Systems Medicine, Tor Vergata University, Rome, Italy; [3]Synaptic Immunopathology Lab, IRCCS San Raffaele Roma, Rome, Italy; [4]Department of Human Sciences and Quality of Life Promotion University of Rome San Raffaele, Rome, Italy; [5]Neuroimmunology Unit, Institute of Experimental Neurology (INSpe), Division of Neuroscience, San Raffaele Scientific Institute, Milan, Italy; [6]European Molecular Biology Laboratory, Mouse Biology Unit, Monterotondo Scalo, Rome, Italy; [7]Department of Molecular Genetics, Weizmann Institute of Science, Rehovot, Israel

Details of EAE Symptoms Evaluation

After EAE induction, animal clinical score (0, healthy; 1, limp tail; 2, ataxia and/or paresis of hindlimbs; 3, paralysis of hindlimbs and/or paresis of forelimbs; 4, tetraparalysis; 5, moribund or death) was recorded daily. All the efforts were made to minimize the number of animals used and their suffering. In particular, when animals experienced hindlimb weakness, moistened food and water were made easily accessible to the animals on the cage floor. Mice with hindlimb paresis received glucose solution by subcutaneous injection or food by gavage during the entire procedure. In the rare presence of a tetraparalyzed animal, death was provided.

Details of Electrophysiological Recordings

The composition of the control ACSF was (in mM): 126 NaCl, 2.5 KCl, 1.2 $MgCl_2$, 1.2 NaH_2PO_4, 2.4 $CaCl_2$, 11 glucose, 25 $NaHCO_3$. MSNs were identified for their morphological and electrophysiological properties [1,2]. To study glutamate-mediated spontaneous excitatory postsynaptic currents (sEPSCs), the recording pipettes were filled with internal solution of the following composition (mM): K^+-gluconate (125), NaCl (10), $CaCl_2$ (1.0), $MgCl_2$ (2.0), 1,2-bis (2-aminophenoxy) ethane-N,N,N,N-tetraacetic acid (BAPTA; 0.5), HEPES (19), GTP (0.3), Mg-ATP (1.0), adjusted to pH 7.3 with KOH. Picrotoxin (50 µM) was added to the perfusing solution to block $GABA_A$-mediated transmission. Synaptic events were stored using PCLAMP (Axon Instruments - Molecular Devices, San Jose, CA, USA) and analysed offline on a personal computer with Mini Analysis 6.0.7 (Synaptosoft, Leonia, NJ, USA) software. The detection threshold of sEPSCs was set at twice the baseline noise. Offline analysis was performed on spontaneous synaptic events recorded during fixed time epochs (1–2 min, three to five samplings), sampled every 5 or 10 min. Only cells that exhibited stable frequencies in control (less than 20% changes during the control samplings) were used for analysis. For kinetic analysis, events with peak amplitude between 5 and 40 pA were grouped, aligned by half-rise time, normalized by peak amplitude and averaged to obtain rise times and decay times.

To lower the miR-142-3p levels, some corticostriatal slices were pre-incubated with LNA anti-miR-142-3p (AAGTAGGAAACACTAC, Exiqon large-scale synthesis; catalog #426804; 0.5 pmol/µl). LNA scramble (ACGTCTATACGCCCA, Exiqon large-scale synthesis; catalog #1990020; 0.5 pmol/µl) was used instead of LNA anti-miR-142-3p as control condition.

1875-6190/23 $65.00+.00 © 2023 Bentham Science Publishers

Details of Confocal Microscopy

All images acquired by the confocal laser-scanner microscope had a pixel resolution of 1024×1024. Z-stack acquisitions (20x objective, zoom 1x with 2 μm interval for a total of 17 steps) were made applying the same intensity and exposure time and large image function that generates a single high-magnification image (capturing 2 images). A 40x and 60x objective (zoom 1x with 1 μm interval for a total of 15 steps) was used to detect TNF double staining with IBA1 and 60x objective (zoom 1x with 1 μm interval for a total of 15 steps) was used to detect CD3. A z-projection image derived from all captured images was produced. All images were processed using ImageJ software and were adjusted for reducing noise by applying smooth and background subtraction as required by the NIH ImageJ. A colocalization mask (mk) was generated on single slices and then Z-projected images were produced to visualise IBA1 and TNF colocalization.

Details of Patient's Recruitment

A cohort of 151 pwMS admitted to the neurological clinic of INM-Neuromed Hospital (Clinically Isolated Syndrome, CIS, n =18; Relapsing-Remitting multiple sclerosis, RRMS, n =108; Progressive multiple sclerosis, PMS, n =25) was included in the study. Details of patients' enrollment and their eligibility criteria were reported in [3]. CSF levels of miR-142-3p and TNF were detected at diagnosis (T0) in all enrolled patients and the following demographic and clinical variables (T0) were considered and analyzed in this study: sex (F/M); age (in years); MS subtype (non-PMS: CIS, RRMS; PMS); disease activity including clinical and/or radiological activity (evaluated by conventional MRI scans, 1.5 Tesla and assessed according to [4]; CSF oligoclonal banding; disease duration, estimated as the number of months from onset to the most recent assessment of disability; clinical disability, assessed by Expanded Disability Status Scale (EDSS); Progression Index (PI = EDSS/disease duration in months); global Age Related Multiple Sclerosis Severity (gARMSS), score obtained by ranking EDSS values on the basis of patient's age at the time of assessment [5]. For details on demographic and clinical characteristics see Table 1.

In 39 patients, an additional 3T MRI scan was performed by using General Electric Signa HDXT Twin Speed MRI equipped with an 8-channel head coil, as in [6]. Structural MRI measures included cortical lesion load and lesion volume and were computed from fluid-attenuated inversion recovery (FLAIR), T2-weighted images (T2-WI) and pre-contrast and post-contrast T1-weighted images (T1-WI) after intravenous gadolinium (Gd) infusion (0.2 ml/kg). For details on demographic and clinical characteristics see Table 2.

REFERENCES

[1] Kreitzer AC. Physiology and pharmacology of striatal neurons. Annu Rev Neurosci. 2009;32:127–47.

[2] Mao M, Nair A, Augustine GJ. A Novel Type of Neuron Within the Dorsal Striatum. Front Neural Circuits. 2019;13:32.

[3] De Vito F, Musella A, Fresegna D, Rizzo FR, Gentile A, Stampanoni Bassi M, et al. MiR-142-3p regulates synaptopathy-driven disease progression in multiple sclerosis. Neuropathol Appl Neurobiol. 2021;

[4] Polman CH, Reingold SC, Banwell B, Clanet M, Cohen JA, Filippi M, et al. Diagnostic criteria for multiple sclerosis: 2010 Revisions to the McDonald criteria. Ann Neurol. 2011;69(2):292–302.

[5] Manouchehrinia A, Westerlind H, Kingwell E, Zhu F, Carruthers R, Ramanujam R, et al. Age Related Multiple Sclerosis Severity Score: Disability ranked by age. Mult Scler. 2017 Dec;23(14):1938–46.

[6] Dolcetti E, Bruno A, Azzolini F, Gilio L, Moscatelli A, De Vito F, et al. The BDNF Val66Met Polymorphism (rs6265) Modulates Inflammation and Neurodegeneration in the Early Phases of Multiple Sclerosis. Genes (Basel). 2022 Feb;13(2).

Supplementary Table 1. List of primers used in qPCR experiments.

Gene	Primers
Hsa-miR-142-3p (QIAGEN)	Cat. YP00204291<br>UGUAGUGUUUCCUACUUUAUGGA
Hsa-miR-204-5p (QIAGEN)	Cat. YP00206072<br>UUCCCUUUGUCAUCCUAUGCCU
miR-142-3p (TaqMan)	Cat. 000464
U6 snRNA Control (TaqMan)	Cat. 001973
Tnf (TaqMan)	Cat. Mm00443258_m1
Aif1, mRNA coding for IBA-1 (TaqMan)	Cat. Mm00479862_1g
Gfap (TaqMan)	Cat. Mn012533033
Cd3e (TaqMan)	Cat. Mm00599684_g1
Actb, mRNA coding for β-actin (TaqMan)	Cat. Mm00607939_s1

Supplementary Table 2. Experimental design and statistical analysis.

Figure	Pre hoc	Post hoc	N / n<br>(Number Of Animals / Samples)
**1 A**	Two-way ANOVA:<br>Interaction F (13,826)=1.522 p=0.1033	Bonferroni multiple comparisons test	N: WT-EAE 32; HE-EAE 29
**1 A'**	Unpaired two-tailed Student's t test:<br>t=2.578, p=0.012		N: WT-EAE 32; HE-EAE 29
**1 B**	One-way ANOVA:<br>F (3, 25) = 17.75, p <0.0001	Tukey multiple comparisons test	N: WT-ctrl 8; WT-EAE 5; HE-ctrl 9; HE-EAE 7
**1C**	One-way ANOVA:<br>(1) F (3, 65) = 8.246, p<0.0001<br>(2) F (3, 65) = 4.805, p=0.0044	Tukey multiple comparisons test	N: WT 4, HE 5<br>n (number of cells): WT-ctrl 17; WT-EAE 10; HE-ctrl 24; HE-EAE 18
**2A**	One-way ANOVA:<br>(1) F (3, 25) = 6.887, p=0.002<br>(2) F (3, 25) = 18.50, p<0.001<br>(3) F (3, 25) = 10.93, p<0.001<br>(4) F (3, 25) = 7.574, p<0.001	Tukey multiple comparisons test	N: WT-ctrl 8; WT-EAE 5; HE-ctrl 9; HE-EAE 7
**2D**	One-way ANOVA:<br>F(2,10) =10.39, p = 0.0036	Tukey multiple comparisons test	N: WT-ctrl=4, WT-EAE=4, HE-EAE=5
**3A**	One-way ANOVA:<br>(1) F (3, 63) = 7.184, p=0.0003<br>(2) F (3, 63) = 4.518, p=0.0062	Tukey multiple comparisons test	N: WT-VEH 3; WT-TNF 3; HE-VEH 4; HE-TNF 3;<br>n (number of cells): WT-VEH 14; WT-TNF 21; HE-VEH 20 HE-TNF 12
**3B**	Unpaired two-tailed Student's t test:<br>t=0.4206, p=0.6775		n (slices): VEH 14; TNF 14<br>N (animals): VEH 2; TNF 2
**3C**	Nonparametric Spearman correlation analysis:<br>r =0.4890, 95% confidence interval=-0.1030 to 0.8252, p (two-tailed)=0.0930		N (number of animals): 13
**4A**	Unpaired two-tailed Student's t test:<br>(1) t=7.396, p<0.0001<br>(2) t=4.416, p<0.0001		N: SCRM-TNF 5; Anti-miR-TNF 4<br>n (number of cells): SCRM-TNF 28; Anti-miR-TNF 20
**4B**	Paired two-tailed Student's t test:<br>(1) Anti-miR-TNF t=2.975, p = 0.0177<br>(2) SCRM-TNF t=0.2111, p = 0.8381		N: SCRM-TNF 5; Anti-miR-TNF 4<br>n (number of cells): SCRM-TNF 28; Anti-miR-TNF 20
**5A**	Unpaired two-tailed Student's t test:<br>(1) t=0.4788, p = 0,6355<br>(2) t=0.3131, p = 0.7563		N: HE-TNF-VEH 3; HE-TNF-AM281 3<br>n (number of cells): HE-TNF-VEH 10; HE-TNF-AM281 20
**5B**	Unpaired two-tailed Student's t test:<br>(1) t=1.503, p = 0.1478<br>(2) t=0.9233 p = 0.3663		N: EAE-HE-VEH 4; EAE-HE-AM281 3<br>n (number of cells): EAE-HE-VEH 14; EAE-HE-AM281 9
**6A**	Nonparametric Spearman correlation analysis:<br>Spearman r: 0.07908, 95% confidence interval:-0.08645 to 0.2403, p(two-tailed) =0.3344		n (number of patients): 151
**6B**	Nonparametric Spearman correlation analysis:<br>r =0.1839, 95% confidence interval=0.02051 to 0.3381, p (two-tailed)=0.0238		n (number of patients): 151
**6C**	Nonparametric Spearman correlation analysis:<br>Spearman r: 0.1998, 95% confidence interval:0.03663 to 0.3526, p(two-tailed) =0.0139		n (number of patients): 151

Figure	Pre hoc	Post hoc	N / n<br>(Number Of Animals / Samples)
**6D**	Nonparametric Spearman correlation analysis:<br>Spearman r: 0.04089, 95% confidence interval:-0.1243 to 0.2039, p(two-tailed) =0.6181		n (number of patients): 151
**6E**	Mann Whitney test<br>(1)U =532, p = 0.0145<br>(2)U =483, p = 0.0303		n (number of patients): 151<br>(l/L 40; l/H 35; h/H 39; h/L 37)
**6F**	Contingency analysis, Chi-square<br>z = 2.139; p =0.0325		n (number of patients): 79<br>(l/L 40; h/H 39)
**7A-A'**	Nonparametric Spearman correlation analysis TNF vs:<br>- lesion load: Spearman r: 0.4737, 95% confidence interval:0.1767 to 0.6917, p(two-tailed) =0.0023<br>- lesion volume: Spearman r: 0.4914, 95% confidence interval:0.1990 to 0.7035, p(two-tailed) =0.0015		n (number of patients): 39
**7B-B'**	Nonparametric Spearman correlation analysis miR-142-3p vs:<br>- lesion load: Spearman r: 0.0319, 95% confidence interval:-0.2954 to 0.3524, p(two-tailed) =0.8472<br>- lesion volume: Spearman r: 0.0506, 95% confidence interval:-0.2781 to 0.3688, p(two-tailed) =0.7596		n (number of patients): 39
**7C-C'**	Mann Whitney test<br>- lesion load: U =26, p = 0.0938<br>- lesion volume: U =22, p = 0.0461		n (number of patients): 21<br>(l/L 14; h/H 7)
